# The rheology of three-phase suspensions at low bubble capillary number

**DOI:** 10.1098/rspa.2014.0557

**Published:** 2015-01-08

**Authors:** J. M. Truby, S. P. Mueller, E. W. Llewellin, H. M. Mader

**Affiliations:** 1 Department of Earth Sciences, Durham University, Durham DH1 3LE, UK; 2 Institute of Geosciences, Johannes Gutenberg University Mainz, 55099 Mainz, Germany; 3 School of Earth Sciences, University of Bristol, Bristol BS8 1RJ, UK

**Keywords:** rheology, particle suspension, bubble suspension, analogue experiments, three phase

## Abstract

We develop a model for the rheology of a three-phase suspension of bubbles and particles in a Newtonian liquid undergoing steady flow. We adopt an ‘effective-medium’ approach in which the bubbly liquid is treated as a continuous medium which suspends the particles. The resulting three-phase model combines separate two-phase models for bubble suspension rheology and particle suspension rheology, which are taken from the literature. The model is validated against new experimental data for three-phase suspensions of bubbles and spherical particles, collected in the low bubble capillary number regime. Good agreement is found across the experimental range of particle volume fraction ($0\leq \phi_{p}≲0.5$) and bubble volume fraction ($0\leq \phi_{b}≲0.3$). Consistent with model predictions, experimental results demonstrate that adding bubbles to a dilute particle suspension at low capillarity increases its viscosity, while adding bubbles to a concentrated particle suspension decreases its viscosity. The model accounts for particle anisometry and is easily extended to account for variable capillarity, but has not been experimentally validated for these cases.

## 1. Introduction

Multiphase suspensions of particles and/or bubbles in a continuous liquid phase are common in nature and industry; examples include magma, oil, concrete, foodstuffs, cosmetics, pharmaceuticals, biological fluids and nanofluids. Characterizing, modelling and controlling the flow of these suspensions requires a constitutive rheological model, encapsulating the viscosity of the suspension as a function of the properties of the suspending liquid, the volume fraction and properties of the suspended phase(s), and the flow conditions.

The rheology of two-phase suspensions (bubbles-in-liquid *or* particles-in-liquid, where the liquid is Newtonian) has been the subject of extensive experimental and theoretical research for more than a century. In recent years, significant advances have been made and two-phase constitutive equations are now available which have been validated against experimental data for a wide range of conditions (see [1] for a recent review). By contrast, considerably less research has been directed at understanding the rheology of three-phase suspensions (where bubbles *and* particles are suspended in a liquid) primarily owing to the complexity of the problem. Phan-Thien & Pham [2] present a theoretical treatment—discussed later in §3—which has been applied in studies of multiphase magma (e.g. [3,4]), but has not been experimentally validated. Experimental investigation of the rheology of three-phase suspensions appears to be confined to studies of bubble- and crystal-bearing magmas (e.g. [4,5]); these experiments and materials are complex and the resulting data are not well-suited to the validation of three-phase rheological models. Constraining three-phase rheology therefore remains an important, yet outstanding, problem in multiphase fluids research.

Here, we build on published two-phase constitutive equations to generate a three-phase model, by using an ‘effective-medium’ method in which the bubble suspension is treated as a continuous medium which suspends the particles; this carries the implicit assumption that the bubbles are small compared with the particles. We validate the model against new experimental data for three-phase suspensions of bubbles and spherical particles in the low-capillarity regime (in which flow is steady and bubble deformation is small).

## 2. Rheology of two-phase suspensions

The rheology of a strictly Newtonian fluid is completely described by its viscosity *μ*. The viscosity is the ratio of the deforming stress and associated strain-rate which, for rheometric flow, is given by $\mu =\tau /\dot{\gamma }=\text{const.}$, where *τ* is the shear stress and $\dot{\gamma }$ is the shear strain-rate. When bubbles or solid particles are added to a Newtonian liquid, the resulting suspension has non-Newtonian rheology. In the simplest case, this means that the ratio of stress and strain-rate is a function of strain-rate and is termed the apparent viscosity $\eta =\tau /\dot{\gamma }=f(\dot{\gamma })$. The viscosity of a suspension is often reported as the relative viscosity *η*_r_, which is the apparent viscosity of the suspension at some strain-rate, normalized by the viscosity of the liquid phase

2.1 $$\eta_{r}=\frac{\eta }{\mu }.$$

In the following subsections, we briefly review the constitutive equations for two-phase suspensions that provide the building blocks for the three-phase rheological model presented later in §3. The subscript ‘b’ refers to bubbles suspensions, the subscript ‘p’ refers to particle suspensions.

### (a) Bubble suspension rheology

When a bubble suspension flows, viscous stresses cause the bubbles to deform. If the flow is ‘steady’ the bubbles reach an equilibrium deformation, which is described by the bubble capillary number

2.2 $$Ca=\lambda \dot{\gamma },$$

where *λ* is the bubble relaxation time [6–8]. The relaxation time describes the characteristic timescale over which the bubble adjusts towards a new equilibrium deformation in response to a change in the strain environment; it is given by

2.3 $$\lambda =\frac{\mu a}{\Gamma },$$

where *a* is the bubble’s equivalent spherical radius and *Γ* is the liquid–gas surface tension. The flow is steady if the condition $\lambda \ll \dot{\gamma }/\ddot{\gamma }$ has been satisfied for time *t*≫*λ* [7].

For steady flow, the relative viscosity *η*_r_b__ of a bubble suspension is given by Rust & Manga [8] and Mader *et al.* [1]

2.4 $$\eta_{r_{b}}\equiv \frac{\eta_{b}}{\mu }=\eta_{r,\infty }+\frac{\eta_{r,0}-\eta_{r,\infty }}{1+{\left((6/5)Ca\right)}^{2}},$$

where *η*_b_ is the apparent viscosity of the bubble suspension, and *η*_r,0_ and $\eta_{r,\infty }$ are, respectively, the relative viscosity of the bubble suspension at low and high *Ca*. For non-dilute suspensions *η*_r,0_ and $\eta_{r,\infty }$ are given by Llewellin & Manga [9] and Mader *et al.* [1]

2.5 $$\eta_{r,0}={\left(1-\phi_{b}\right)}^{-1}$$

and

**Figure 1.**
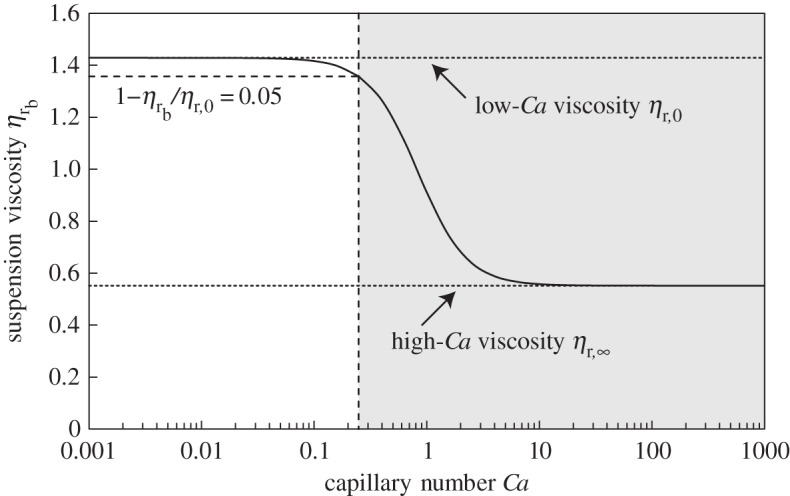
Normalized bubble suspension viscosity *η*_r_b__ as a function of capillary number *Ca* for *ϕ*_b_=0.3 (solid line). Short-dashed lines show the asymptotic values of *η*_r_b__ at low and high capillary number. Long-dashed line shows the capillary number that we define as the upper limit of the low-capillarity region (*Ca*≥0.248 for *ϕ*_b_=0.3); we discard experimental data collected above this value, in the shaded region, as discussed in §4*c*.

2.6 $$\eta_{r,\infty }={\left(1-\phi_{b}\right)}^{5/3},$$

where *ϕ*_b_ is the bubble volume fraction. These expressions, which reduce in the dilute limit (as $\phi_{b}\to 0$) to the well-known theoretical models of Taylor [10] (*η*_r,0_=1+*ϕ*_b_) and Mackenzie [11] $\left(\eta_{r,\infty }=1-5\phi_{b}/3\right)$, show that bubbles increase suspension viscosity at low *Ca* and decrease suspension viscosity at high *Ca*. Equation (2.4) is plotted in figure 1, which demonstrates that the transition between the asymptotic viscosity regions at low and high capillarity occurs over a fairly narrow range of *Ca*, centred on *Ca*≈1. Consequently, equations (2.5) and (2.6) can be used to calculate bubble suspension viscosity for all *Ca*, except over the narrow transitional region; we define an approximate upper bound to the low *Ca* region later in §4*c*.

Equations (2.2)–(2.6) are relevant for monodisperse bubble suspensions at low and moderate bubble volume fractions ($\phi_{b}≲0.5$) [7]. Most bubble suspensions are polydisperse to some extent, resulting in a range of bubble relaxation times; hence also a range of capillarities for a given strain-rate. In this work, we restrict our analysis to suspensions in the low-capillarity limit, where equation (2.5) is sufficient to describe the viscosity of a bubble suspension regardless of its bubble size distribution. For the more general case of intermediate capillarity, a more sophisticated approach is required, in which the contribution of each bubble size fraction to the viscosity of the bulk suspension is linearly superposed; this approach is described in detail in Mader *et al.* [1]. Bubble suspensions are visco-elastic even when dilute, and elastic behaviour becomes more pronounced as bubble volume fraction increases; visco-elastic rheology is neglected in this work because elastic behaviour is not manifest in steady flow [7].

### (b) Particle suspension rheology

Particle suspensions commonly show non-Newtonian behaviour when non-dilute, including shear-thinning (e.g. [12]), shear-thickening (e.g. [13,14]) and non-zero normal stress differences (e.g. [15, 16]). When shear-thinning behaviour is observed, the rheology of a particle suspension is often described using the model of Herschel & Bulkley [17]

2.7 $$\tau =\tau_{0}+K{\dot{\gamma }}^{n},$$

where *τ*_0_ is the yield stress, *K* is the consistency and *n* is the flow index (*n*<1 when the suspension is shear-thinning). The yield stress is non-zero only for highly concentrated suspensions, hence it is often neglected, reducing equation (2.7) to a power-law [18]

2.8 $$\eta_{p}=K{\dot{\gamma }}^{n-1},$$

where *η*_p_ is the apparent viscosity of the suspension. Although in common usage (e.g. [4,19,20]), this approach has the limitation that the consistency has fractional units of Pa s^*n*^ and is therefore not amenable to non-dimensionalization when *n*≠1; this issue is discussed in detail in Mader *et al.* [1] and Mueller *et al.* [20]. In this work, we address this limitation by introducing a characteristic timescale *t*_c_ of a shear-thinning suspension, against which the strain-rate can be non-dimensionalized, giving

2.9 $$\eta_{p}=\eta_{*}{\left(t_{c}\dot{\gamma }\right)}^{n-1},$$

where η_*_ is a ‘reference viscosity’ of the suspension — i.e. the apparent viscosity at strain-rate $\dot{\gamma }=1/t_{c}$. No satisfactory microphysical explanation for shear-thinning has yet been proposed for suspensions of the sort considered in this work, in which the particles are not subject to Brownian motion (high Peclet number), are strongly coupled to the flow (low Stokes number) and in which inertial effects can be neglected (low particle Reynolds number) [19]. Consequently, there is no physical model from which *t*_c_ can be computed *a priori*. However, Mueller *et al.* [19,20] find empirically that the theoretical model of Maron & Pierce [21]

2.10 $$\eta =\mu {\left(1-\frac{\phi_{p}}{\phi_{m}}\right)}^{-2}$$

accurately captures the rheology of diverse particle suspensions (with variable *ϕ*, *μ* and particle aspect ratio) when the consistency is identified with the viscosity; i.e. under the assumption *K*≡*η*. This is equivalent to finding that the characteristic timescale *t*_c_=1 *s*, and making the identity η_*_≡*η* in equations (2.9) and (2.10). This allows us to link these two equations while maintaining strict dimensional consistency, giving

2.11 $$\eta_{r,*}={\left(1-\frac{\phi_{p}}{\phi_{m}}\right)}^{-2},$$

where we define *η*_r,*_ as the relative reference viscosity

2.12 $$\eta_{r,*}\equiv \frac{\eta_{*}}{\mu }.$$

We propose that this approach is a useful improvement over that adopted by Mueller *et al.* [19,20], Vona *et al.* [4] and Mader *et al.* [1], which included the pragmatic, but inexact, non-dimensionalization *K*_r_=*K*/*μ*. Numerically, the values of *K*_r_ and *η*_r,*_ are identical when *t*_c_=1 *s* (as is indicated empirically) so the results of those earlier studies can be transferred directly into this new framework.

The maximum packing fraction in equations (2.10) and (2.11) is a function of particle shape and roughness; Mader *et al.* [1] give the following equation for *ϕ*_m_:

2.13 $$\phi_{m}=\phi_{m_{1}}\exp\left[-\frac{{\left(\log_{10}r_{p}\right)}^{2}}{2b^{2}}\right],$$

where *r*_p_ is the particle aspect ratio. For smooth particles *ϕ*_m_1__=0.66 and *b*=1.08, and for rough particles *ϕ*_m_1__=0.55 and *b*=1.00; these values are empirically determined.

Mueller *et al.* [19] report that the flow index *n* for a particle suspension is a function of the particle volume fraction *ϕ*_p_ and the particle aspect ratio. They present a purely empirical relationship

2.14 $$n=1-0.2r_{p}{\left(\frac{\phi_{p}}{\phi_{m}}\right)}^{4},$$

which is valid for *ϕ*_p_/*ϕ*_m_≤0.8.

## 3. A model for the rheology of three-phase suspensions

Equation (2.11) gives the relative viscosity *η*_r,*_ of a suspension of particles in a liquid with viscosity *μ*. If we suppose that the particles are instead suspended in a bubble suspension with viscosity *η*_b_ (i.e. we treat the bubble suspension as an ‘effective medium’) we obtain

3.1 $$\frac{\eta_{*}}{\eta_{b}}={\left(1-\frac{\phi_{p}}{\phi_{m}}\right)}^{-2}.$$

Treating the bubble suspension as the continuous phase carries the implicit assumption that the bubbles should be small compared with the particles. At low bubble capillarity, from equations (2.4) and (2.5), we have *η*_b_=*μ*(1−*ϕ*_b_)^−1^; hence

3.2 $$\eta_{r,*}={\left(1-\phi_{b}\right)}^{-1}{\left(1-\frac{\phi_{p}}{\phi_{m}}\right)}^{-2}.$$

At high bubble capillarity, equation (2.6) would take the place of equation (2.5); while for intermediate capillarity, equation (2.4) would take its place, and polydispersity would have to be explicitly accounted for (see §2*a*).

This effective medium method has been used elsewhere in rheological models. Of most relevance, Phan-Thien & Pham [2] use the approach to derive an equation for the viscosity of three-phase suspensions of bubbles and particles that is similar to the model we derive above, but contains a different expression for the particle suspension contribution: (1−*ϕ*_p_)^−5/2^, which they derive using a differential method. Although they do consider a maximum packing fraction in some variants of their model, their implicit solutions reduce to exact equations only under restrictive conditions, e.g. *ϕ*_p_∼*ϕ*_m_ or *ϕ*_m_=1. Consequently, the treatment of the contribution of the particles to the suspension rheology in our formulation is a significant improvement over that of Phan-Thien & Pham [2].

### (a) Defining volume fractions in three-phase suspensions

Particle volume fraction and gas volume fraction are unambiguously defined for two-phase suspensions; however, care must be taken to define them appropriately for three-phase suspensions. In our model formulation above, we treat the bubble suspension as the effective medium, hence, the appropriate definitions are

3.3 $$\phi_{b}=\frac{V_{b}}{V_{l}+V_{b}}$$

and

3.4 $$\phi_{p}=\frac{V_{p}}{V_{l}+V_{b}+V_{p}},$$

where *V*_*l*_, *V*_b_ and *V*_p_ are the respective volumes of the liquid, bubble and particle phases.

For many three-phase applications, we are interested in characterizing how the rheology of a suspension of particles changes as bubbles are added to it (or, equivalently, as bubbles grow within it). For example, a magma that contains solid crystals may be bubble-free at depth, but become increasingly bubble-rich during ascent. In this case, it is more intuitive to define a particle volume fraction and bubble volume fraction as follows:

3.5 $$\phi_{b}^{*}=\frac{V_{b}}{V_{l}+V_{b}+V_{p}}$$

and

3.6 $$\phi_{p}^{*}=\frac{V_{p}}{V_{l}+V_{p}}.$$

In this formulation, the particle volume fraction does not change from its initial value as bubbles grow, and the bubble volume fraction reflects the value that would be measured by applying Archimedes’ principle on the bulk sample. The different formulations for volume fractions are simply related

3.7 $$\phi_{b}=\frac{\phi_{b}^{*}}{1-\phi_{p}^{*}(1-\phi_{b}^{*})}$$

and

3.8 $$\phi_{p}=\phi_{p}^{*}(1-\phi_{b}^{*}),$$

allowing the three-phase model (equation (3.2)) to be applied when it is ϕ*_b_ and ϕ*_p_ that are known.

In the following sections, we work with both of these definitions, since equations (3.3) and (3.4) underpin the model formulation, while equations (3.5) and (3.6) are more natural for some applications of the model, and aid physical insight. A further volume fraction of interest is the fraction of the total volume that is made up of suspended bubbles and/or particles—the total suspended fraction:

3.9 $$\phi_{s}=\frac{V_{b}+V_{p}}{V_{l}+V_{b}+V_{p}}.$$

## 4. Experiments

### (a) Samples

Three-phase samples were prepared by adding spherical glass beads (Potters Ballotini; density 2448 kg m^−3^, size fraction 63–125 μm) to a sugar syrup (Tate & Lyle Golden Syrup; density 1438 kg m^−3^ and surface tension 0.08 N m^−1^ [7]) and aerating with a domestic electric whisk. The rheology of the pure syrup was determined individually for each sample batch and found to be strictly Newtonian; measured viscosities were in the range 55.68≤μ≤61.69 Pa s at 20°C (presented later in data table 1). Particle volume fraction was controlled by adding a known mass of beads to a known mass of syrup (typically equating to 100–150 ml) to prepare sample suites of similar initial (bubble-free) particle volume fraction *ϕ*_p_=0.05, 0.1, 0.2, 0.3, 0.4 and 0.5. Bubble volume fraction was varied by adjusting the duration and speed of whisking, and suspension temperature. Errors in particle volume fraction and bubble volume fraction are ±3% and ±5%, respectively.

**Table 1.** Experimental data for each sample, to three significant figures, and 1 s.d. errors where appropriate. Column $a_{\max}$ gives the largest bubble measured for each sample, where asterisk (*) indicates that no bubble size distribution was measured, and an average value was used instead. Column ${\dot{\gamma }}_{\max}$ gives the maximum strain-rate that satisfies equation (4.2), and *N*_data_ are the number of stress–strain-rate data points that remain after filtering.

sample	*ϕ*_p_±3%	ϕ*_p_±5.83%	*ϕ*_b_±5.83%	ϕ*_b_±5%	μ (Pa s)	$a_{\max}$ (μm)	η_*_ (Pa s) ± (error)	*n*± (error)	${\dot{\gamma }}_{\max}$ (s^−1^)±2%	*N*_data_
3P-3	0.102	0.120	0.168	0.151	61.7	455	117 (1)	0.944 (0.004)	0.646	18
3P-5	0.088	0.113	0.237	0.216	61.7	245	118 (1)	0.916 (0.005)	1.15	15
3P-6	0.113	0.120	0.058	0.051	61.7	177	106 (1)	0.979 (0.003)	3.43	32
3P-7	0.102	0.105	0.033	0.030	61.7	122	89.2 (0.4)	0.991 (0.003)	5.73	38
3P-8	0.192	0.207	0.085	0.068	61.7	129	153 (1)	0.965 (0.003)	3.14	36
3P-9	0.165	0.207	0.244	0.204	61.7	*160	159 (1)	0.914 (0.003)	1.44	26
3P-10	0.164	0.207	0.247	0.206	61.7	234	175 (2)	0.935 (0.003)	0.903	24
3P-11	0.177	0.216	0.220	0.181	61.7	144	176 (1)	0.938 (0.003)	1.57	28
3P-12	0.190	0.212	0.127	0.103	61.7	116	143 (1)	0.965 (0.003)	2.60	32
3P-13	0.169	0.212	0.245	0.204	61.7	*160	163 (2)	0.930 (0.003)	1.36	26
3P-14	0.272	0.302	0.135	0.098	61.7	103	235 (1)	0.959 (0.003)	2.28	38
3P-15	0.244	0.303	0.256	0.194	61.7	123	245 (2)	0.908 (0.003)	1.62	32
3P-17	0.274	0.302	0.130	0.094	58.1	*160	234 (2)	0.956 (0.003)	1.59	32
3P-18	0.289	0.306	0.075	0.053	58.1	*160	224 (2)	0.972 (0.003)	2.10	36
3P-19	0.331	0.409	0.287	0.192	58.1	*160	512 (4)	0.845 (0.002)	0.834	34
3P-20	0.338	0.405	0.249	0.165	58.1	150	500 (4)	0.838 (0.002)	0.963	36
3P-21	0.368	0.406	0.147	0.093	58.1	142	498 (3)	0.917 (0.002)	1.07	38
3P-22	0.385	0.405	0.078	0.048	58.1	120	491 (3)	0.937 (0.002)	1.06	38
3P-23	0.000	0.000	0.213	0.213	58.1	229	76.6 (0.7)	0.926 (0.004)	1.72	21
3P-24	0.000	0.000	0.054	0.054	58.1	*160	60.5 (0.4)	0.974 (0.003)	5.30	30
3P-25	0.000	0.000	0.075	0.075	58.1	173	65.4 (0.4)	0.970 (0.004)	3.27	26
3P-26	0.000	0.000	0.095	0.095	58.1	129	63.6 (0.4)	0.959 (0.003)	4.29	28
3P-27	0.000	0.000	0.123	0.123	55.7	136	60.7 (0.4)	0.953 (0.003)	3.58	26
3P-28	0.000	0.000	0.275	0.275	55.7	239	74.4 (0.7)	0.920 (0.004)	1.49	20
3P-29	0.463	0.505	0.157	0.085	55.7	147	1490 (20)	0.771 (0.004)	0.124	38
3P-30	0.042	0.059	0.302	0.290	55.7	99.9	96.5 (0.6)	0.888 (0.003)	3.11	27
3P-31	0.048	0.062	0.240	0.228	55.7	110	97.0 (0.7)	0.919 (0.003)	3.09	28
3P-32	0.057	0.065	0.130	0.123	55.7	150	90.6 (0.6)	0.928 (0.009)	2.84	18
3P-33	0.060	0.065	0.090	0.085	55.7	*160	77.6 (0.6)	0.963 (0.010)	3.57	20
3P-35	0.050	0.050	0.000	0.000	55.9	—	67.4 (0.5)	0.994 (0.006)	7.53	38
3P-36	0.100	0.100	0.000	0.000	55.9	—	81.1 (0.6)	0.994 (0.006)	6.25	38
3P-37	0.200	0.200	0.000	0.000	55.9	—	125 (1)	0.993 (0.006)	4.05	38
3P-38	0.300	0.300	0.000	0.000	55.9	—	222 (1)	0.988 (0.006)	1.36	38
3P-39	0.400	0.400	0.000	0.000	55.9	—	540 (4)	0.955 (0.006)	0.924	38
3P-40	0.500	0.500	0.000	0.000	55.9	—	2260 (40)	0.818 (0.004)	0.083	38
3P-41	0.431	0.508	0.266	0.151	57.0	149	1330 (20)	0.676 (0.002)	0.111	27
3P-42	0.412	0.499	0.295	0.174	57.0	132	1020 (10)	0.689 (0.002)	0.164	27
3P-43	0.449	0.496	0.173	0.095	57.0	133	1280 (10)	0.809 (0.002)	0.177	30
3P-44	0.460	0.497	0.141	0.076	57.0	96.5	1370 (10)	0.803 (0.002)	0.302	38

**Figure 2.**
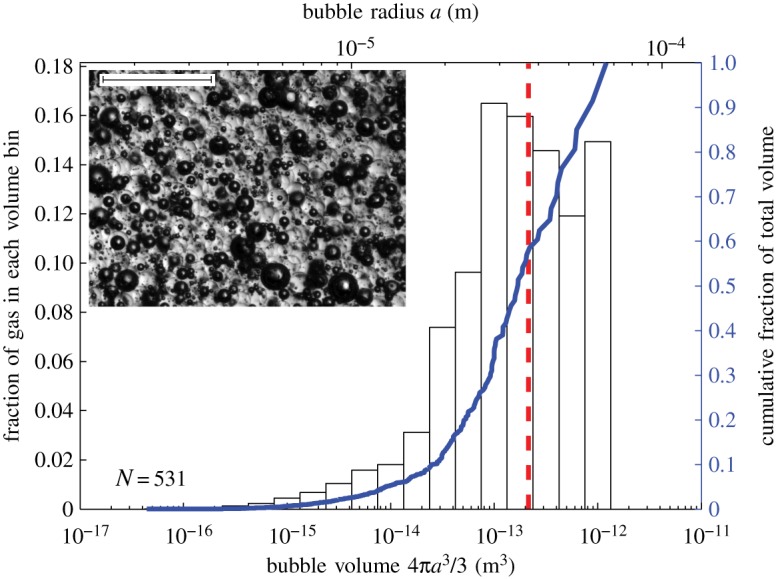
Bubble size distribution for sample 3P-42 (*ϕ*_p_=0.42 and *ϕ*_b_=0.29). Histogram shows the fraction of the total bubble volume in the sample represented in each volume bin. Solid line shows the cumulative fraction of the total bubble volume. Dashed line is the volume-mean-radius 〈*a*〉∼37 μm (see §4*c*). Inset shows photomicrograph of sample, in which dark-rimmed spheres are bubbles, and glass beads are light and translucent. Scale bar, 500 μm. (Online version in colour.)

Of the resulting three-phase suspension, about 60 ml was used for rheometric analysis, and a small amount was imaged with a Zeiss SteREO V.8 stereomicroscope. With the remaining sample material, the bubble volume fraction ϕ*_b_ was determined by measuring its weight and its volume in a 100 ml measuring cylinder. The bubble size distribution of each sample was determined using the image analysis software JMicroVision. A photomicrograph of a typical sample is presented in figure 2, along with its bubble size distribution.

### (b) Rheometry

Rheometric data were collected using a ThermoScientific Haake MARS II rheometer with Z40DIN concentric cylinder sensor geometry (rotor diameter 40.0 mm, cup diameter 43.4 mm, gap width 1.7 mm). A standard flow-curve determination consisted of a 20-step ‘up ramp’ of incrementally increasing shear stress *τ* up to a maximum value of 500 Pa (‘controlled-stress mode’), followed by a 20-step ‘down ramp’. At each stress step, the rheometer recorded the corresponding strain-rate $\dot{\gamma }$ once it had reached equilibrium flow conditions. To ensure equilibrium starting conditions for each test, flow curve determinations were preceded by a 4 min, continuous 0–150–0 Pa stress ramp as pre-shear treatment (following [19]). All experiments were performed at 20°C; the precision of the stress and strain-rate measurements is estimated at ±2%.

The densities of the particles and the suspending liquid are not well-matched in our experiments so settling must be considered; similarly, the bubbles are prone to buoyant rise (‘creaming’). The concentric cylinder sensor geometry was chosen because it is relatively insensitive to effects of settling and creaming compared with, say, a parallel plate geometry, because particles and bubbles move vertically past the sensor, rather than accumulating in a layer against it. From Stokes’ law, we can compute that the time required for an isolated particle or bubble in a dilute suspension to fall or rise the full length of the sensor is more than 120 h for the largest particle and around 1.5 h for the largest bubble; for a concentrated suspension, it is much longer because settling and creaming are hindered. Since total runtimes for our rheometric experiments are much shorter for each sample (the mean experiment duration was 5 min, maximum 12 min), the development of spatial gradients in the particle and bubble volume fractions is considered negligible.

### (c) Data analysis

**Figure 3.**
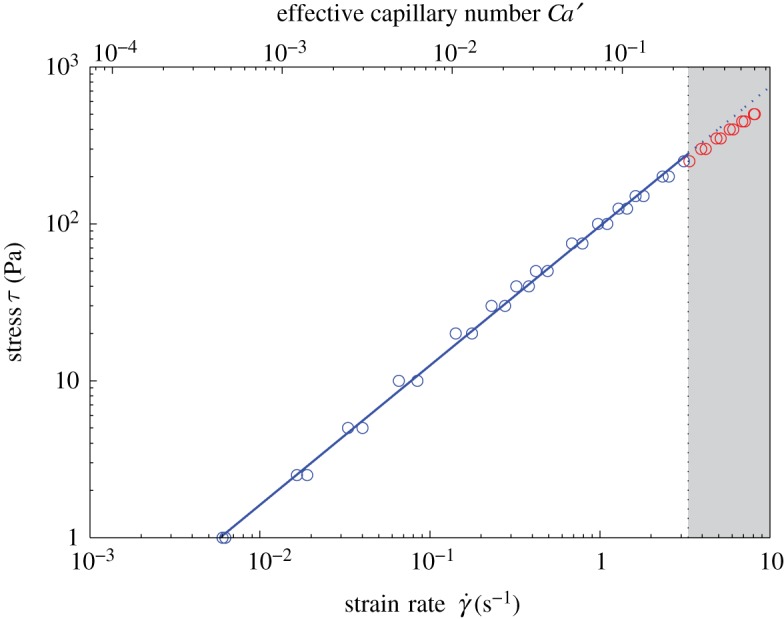
Flow curve of shear stress *τ* against shear strain-rate $\dot{\gamma }$ for sample 3P-30 with *ϕ*_b_=0.30 and *ϕ*_p_=0.04. Datapoints are collected during both the up-ramp and down-ramp (§4*b*). Datapoints are discarded (shaded region) for *Ca*′ (defined in §4*c*) greater than the upper bound of the low-capillarity region, defined according to equation (4.2); for *ϕ*_b_=0.30, this is *Ca*′≥0.248. Solid line is the best fit of equation (4.3) to the remaining datapoints, giving *η*_r,*_=1.74 and *n*=0.89; dashed line is the fit extended into the discarded region. (Online version in colour.)

Our rheometric experiments yield flow curves of applied shear stress *τ* against resultant shear strain-rate $\dot{\gamma }$; an example is shown in figure 3. For some samples, the highest experimental strain-rates are sufficient that the bubbles cannot be assumed to be in the low-capillarity regime. We filter data to remove these datapoints by calculating an upper bound on the low-capillarity region for each sample as follows. For three-phase suspensions, the effective strain-rate in the bubbly effective medium ${\dot{\gamma }}^{\prime }$ is higher than the bulk strain-rate $\dot{\gamma }$, because the solid particles cannot accommodate strain through internal shearing. The effective strain-rate is approximately given by ${\dot{\gamma }}^{\prime }=\dot{\gamma }/(1-\phi_{p}/\phi_{m})$; this is a conservative estimate because it does not account for the accommodation of shear strain through solid-body rotation of the particles. A typical bubble radius for each sample may be calculated as the volume-mean-radius $\langle a\rangle =\sum a^{4}/\sum a^{3}$ where summation is over all measured bubbles in that sample (following [1]). We adopt the more conservative criterion of calculating the capillary number using the radius of the largest bubble measured $a_{\max}$. Putting these values into equations (2.2) and (2.3), we obtain an equation for the effective capillary number: $Ca^{\prime }=\mu a_{\max}{\dot{\gamma }}^{\prime }/\Gamma$. We define the low-capillarity region on the basis of the mismatch between the viscosity calculated from equation (2.5) (the low-capillarity asymptotic viscosity), and from equation (2.4) (which is valid for all capillary numbers). We set the upper bound of the low-capillarity region as the value of *Ca*′ for which the mismatch reaches 5%; i.e. for low capillarity

4.1 $$\frac{\eta_{r,0}-\eta_{r_{b}}}{\eta_{r,0}}<5\%$$

or equivalently, from equation (2.4)

4.2 $$Ca^{\prime }<\sqrt{\frac{5}{144(0.95-{\left(1-\phi_{b}\right)}^{8/3})}}.$$

The 5% threshold is chosen to be in line with experimental error. After filtering, all flow curves comprise at least 15 datapoints. Based on this conservative criterion, the discrepancy (equation (4.1)) for a bubble with volume-mean-radius is never greater than 1%.

We determine the yield stress *τ*_0_, consistency *K* and flow index *n* for each sample by fitting the Herschel–Bulkley model (equation (2.7)) to each filtered flow curve (figure 3) and determine errors using the bootstrapping method presented in the electronic supplementary material. For all samples, the yield stress is found to be either small and negative (which is unphysical) or positive, but within 2*σ* error of zero; hence, yield stress can be neglected and equation (2.7) can be expressed as the simple power law relationship given in equation (2.9). This is consistent with the experimental results of Mueller *et al.* [19], who found that yield stress is negligible for $\phi_{p}/\phi_{m}≲0.8$. This allows us to fit for η_*_ and *n* in log-space using the relationship

4.3 $$\log\tau =\log\eta_{*}+n\log t_{c}\dot{\gamma },$$

which avoids biasing the fit to large values of *τ* and $\dot{\gamma }$. As discussed in §2*b*, we assume *t*_c_=1 *s*, based on previous experimental work [19,20]. The reference viscosity η_*_ that we determine is normalized by the viscosity of the syrup μ to give the relative reference viscosity *η*_r,*_ (equation (2.12)), hereafter referred to as the relative viscosity.

## 5. Results

**Figure 4.**
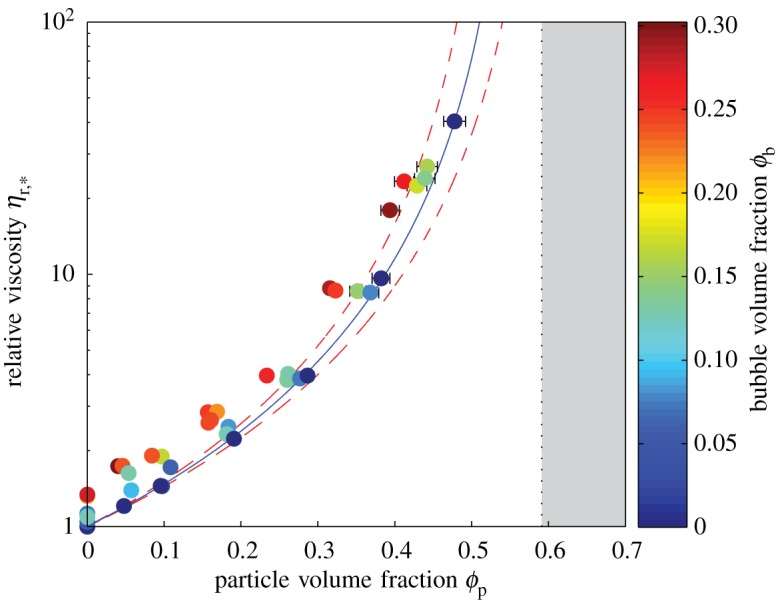
Relative viscosity *η*_r,*_ against particle volume fraction *ϕ*_p_ for three-phase suspensions; circles are shaded according to bubble volume fraction *ϕ*_b_. Solid curve is the best fit of the Maron–Pierce equation (2.11) to data from bubble-free suspensions (*ϕ*_b_=0, dark circles), giving *ϕ*_m_=0.593±0.018. Shaded area shows the region where *ϕ*>*ϕ*_m_. Dashed curves show the 95% confidence limits for *ϕ*_m_ based on the potential error in the data fitting technique, as presented in the electronic supplementary material. Error bars (1*σ*) are shown when larger than the data points. (Online version in colour.)

**Figure 5.**
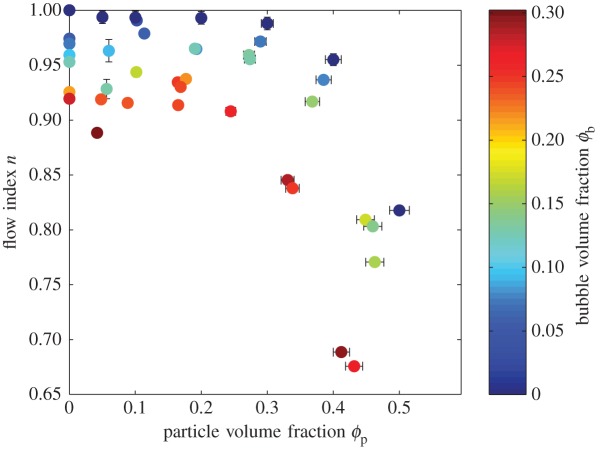
Flow index *n* against particle volume fraction *ϕ*_p_ for three-phase suspensions; circles are shaded according to bubble volume fraction *ϕ*_b_. Error bars (1*σ*) are shown when larger than the data points. (Online version in colour.)

Experimental data are presented in table 1. Results for relative viscosity are presented in figure 4, which plots *η*_r,*_(*ϕ*_p_), with datapoints coloured according to *ϕ*_b_. Similarly, experimental results for the flow index are presented in figure 5, which plots *n*(*ϕ*_p_), with datapoints coloured according to *ϕ*_b_.

It is useful at this stage to confirm that the data for the two-phase end members (bubble-free particle suspension and particle-free bubble suspension) adhere to the relevant two-phase constitutive equations. The solid curve in figure 4 represents the best fit of equation (2.11) (in which the only free parameter is *ϕ*_m_) to data for bubble-free particle suspensions (*ϕ*_b_=0). We find an excellent fit for *ϕ*_m_=0.593, with *R*^2^=1.00; this value of the maximum packing fraction is slightly lower than the value of *ϕ*_m_=0.633 quoted by Mueller *et al.* [19,20] for suspensions of monodisperse spheres, but we note that the 2*σ* errors of the two estimates overlap. Both of these values are lower than the value of *ϕ*_m_=0.66 calculated from equation (2.13) with *r*_p_=1. We propose that equation (2.13) overestimates *ϕ*_m_ for nearly spherical particles because it is based on a fit to data that assumes a Gaussian relationship between *ϕ*_m_ and *r*_p_. Studies of non-sheared particle packs have reported that the maximum packing fraction is actually highest for slightly non-spherical aspect ratios [22,23]; consequently, the *ϕ*_m_(*r*_p_) curve dips around *r*_p_=1 leading to the overestimate given by equation (2.13).

**Figure 6.**
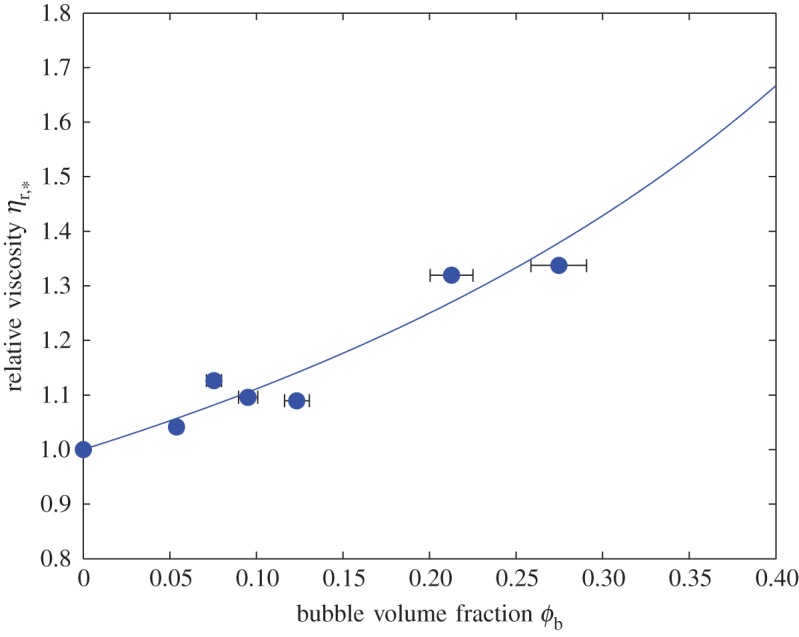
Relative viscosity *η*_r,*_ against bubble volume fraction *ϕ*_b_ for particle-free (two-phase) bubble suspensions. The solid curve is equation (2.5). Error bars (1*σ*) are shown when larger than the data points. (Online version in colour.)

Figure 6 plots two-phase data for the relative viscosity of particle-free bubble suspensions, i.e. *η*_r,*_(*ϕ*_b_) with *ϕ*_p_=0. The solid curve is equation (2.5), which gives a good fit to data, with *R*^2^=0.87. Figures 4 and 6, therefore, demonstrate the validity of the two-phase constitutive models (equations (2.5) and (2.11)) on which we build our three-phase model.

## 6. Discussion

### (a) Reference viscosity

In figure 4, all suspensions that contain bubbles have a higher reference viscosity than a two-phase particle suspension with the same particle volume fraction. Conceptually, this is equivalent to saying that, for a given particle suspension, the viscosity increases if some of the suspending liquid is replaced with bubbles. This is intuitive, because bubbles in the low-capillarity regime increase suspension viscosity.

**Figure 7.**
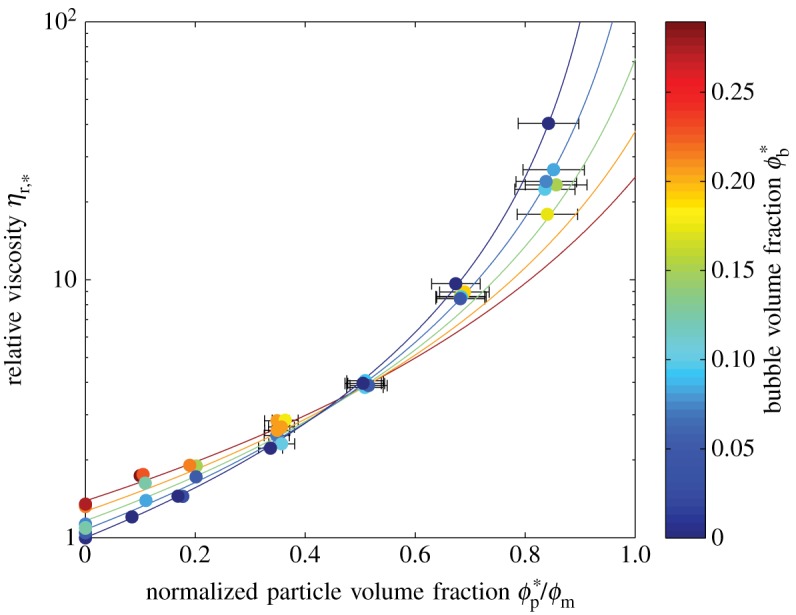
Three phase data from figure 4 recast in terms of ϕ*_b_ and ϕ*_p_. Solid lines are the three-phase model (equation (3.2)) contoured in ϕ*_b_. Error bars (1*σ*) are shown when larger than the data points. (Online version in colour.)

Recasting our data in terms of ϕ*_b_ and ϕ*_p_ yields figure 7. It is evident from this plot that the effect of *adding* bubbles to a particle suspension (or growing bubbles in a particle suspension) depends upon the initial particle volume fraction ϕ*_p_. For dilute particle suspensions ($\phi_{p}^{*}≲0.25$), adding bubbles increases the suspension viscosity; whereas, for more concentrated particle suspensions ($\phi_{p}^{*}≳0.25$), adding bubbles decreases suspension viscosity.

**Figure 8.**
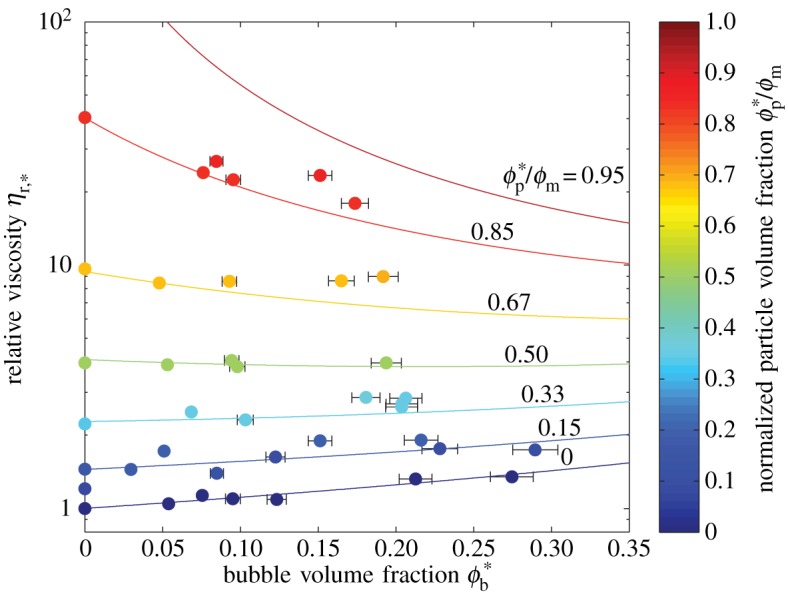
Relative viscosity *η*_r,*_ against ϕ*_b_. Datapoints are shaded according to ϕ*_p_/*ϕ*_m_. Solid curves are the three-phase model (equation (3.2)) for experimental values of ϕ*_p_/*ϕ*_m_ appropriate for each suite of samples, contoured according to the same scale. Error bars (1*σ*) are shown when larger than the data points. (Online version in colour.)

These relationships are more clearly demonstrated by figure 8. The plot shows that the rheology of a particle suspension becomes increasingly sensitive to the addition of a small volume fraction of bubbles as its particle volume fraction approaches the maximum packing fraction. The physical explanation for this behaviour is straightforward and relies on two competing processes. As discussed above, the addition of low-capillarity (i.e. spherical) bubbles to a fluid increases its viscosity. For dilute particle suspensions, this is the dominant trend, hence our data show an increase in reference viscosity with increasing bubble content for $\phi_{p}^{*}≲0.25$. Opposing this is a ‘dilution’ effect, in which the addition of bubbles to a suspension of particles moves the particles further apart; this decreases the particle volume fraction *ϕ*_p_, reduces the impact that particle–particle interactions have on suspension rheology and reduces suspension viscosity. This process dominates for concentrated particle suspensions; indeed, because the Maron–Pierce relationship is a power law, the higher the initial particle volume fraction, the greater the impact the same dilution with bubbles will have.

**Figure 9.**
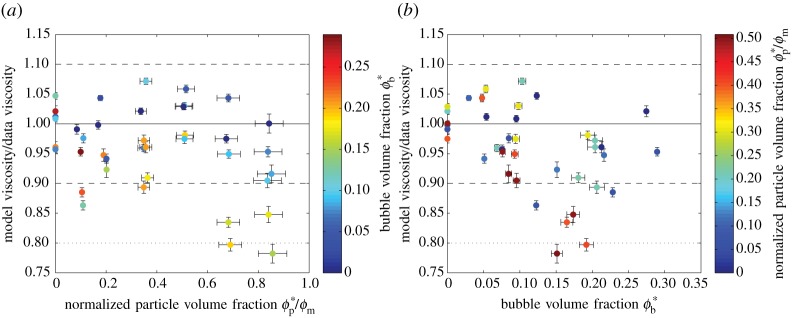
Comparison between *η*_r,*_ predicted by the model (equation (3.2)) and *η*_r,*_ calculated from experimental data, against (*a*) ϕ*_p_/*ϕ*_m_, shaded for ϕ*_b_, and (*b*) ϕ*_b_, shaded for ϕ*_p_/*ϕ*_m_. Error bars (1*σ*) are shown when larger than the data points. The solid line indicates a perfect match between model and data; the dashed and dotted lines, respectively, indicate ±10% and ±20% discrepancy. (Online version in colour.)

It is also clear from these figures that the data agree well with the three-phase model that we propose (equation (3.2)). The model predicts the relative viscosity to within ±20% for all but two of the samples, and within ±10% for the majority (figure 9). Furthermore, there is no strong systematic trend relating the discrepancy to either ϕ*_b_ or ϕ*_p_. We note that the close agreement between model and data is found despite the fact that some samples violate the model assumption that bubbles are small compared with particles (§3). In all samples, the majority (by number) of the bubbles are smaller than the particles and so it makes sense to choose the bubble suspension as the effective medium. However, the average bubble radius is between 0.20 and 0.97 times the average particle radius and the bubble volume-mean-radius (§4*c*) is between 0.57 and 2.4 times the particle volume-mean-radius indicating that the ‘typical’ bubble in many samples is comparable in size to the particles.

The largest discrepancy between our model and data occurs when both ϕ*_p_ and ϕ*_b_ are large: our model tends to underpredict the reference viscosity in such samples. This discrepancy is probably caused by bubble–particle interactions, which are not captured in our simple approach of combining two-phase equations. Further work is needed to formulate a model that captures such interactions.

For a given bubble and particle volume fraction, it is useful to know whether the addition of further bubbles (or the growth of existing bubbles) will result in an increase or decrease in viscosity. An analysis of this scenario is presented in the electronic supplementary material.

### (b) Flow index

**Figure 10.**
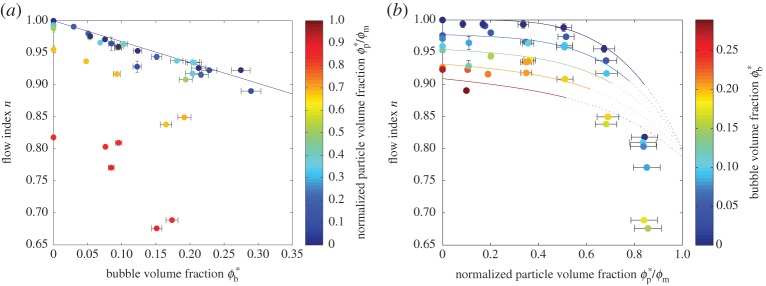
Flow index *n* against (*a*) ϕ*_b_, shaded according to ϕ*_p_/*ϕ*_m_, and (*b*) ϕ*_p_/*ϕ*_m_, shaded according to ϕ*_b_. Error bars (1*σ*) are shown when larger than the data points. (*a*) The solid line shows the linear relationship between *n* and ϕ*_b_ for ϕ*_p_=0 (equation (6.1)). (*b*) The curves, contoured according to ϕ*_b_, plot equation (6.2); curves are solid over the range of particle volume fractions for which the total suspended fraction *ϕ*_*s*_<0.5, and are dotted outside this range. (Online version in colour.)

Reference viscosity provides only a partial description of the rheology of a shear-thinning suspension; for a practical rheological model, the flow index *n* is also required (equation (2.9)). Figure 10 re-presents the flow index data shown in figure 5, plotting them against ϕ*_b_ and ϕ*_p_/*ϕ*_m_.

Figure 10*a* shows clearly that shear thinning is observed for all suspensions, even those containing only bubbles. Our data indicate a linear relationship between *n* and *ϕ*_b_ for particle-free suspensions, in the low-capillarity regime, such that shear thinning becomes more pronounced as bubble volume fraction increases:

6.1 $$n=1-0.334\phi_{b}.$$

(Note that $\phi_{b}=\phi_{b}^{*}$ when *ϕ*_p_=0.) Bubble suspensions are known to be strongly shear thinning in the range 0.1≲Ca≲10 but, at lower and higher capillary number, current models predict that shear thinning is negligible (see figure 1 and §2*a*), which makes this result surprising. One potential explanation is that, despite our data filtering methodology (§4*c*), there are a small number of very large bubbles in the samples, larger than those measured in our sample images, which have a correspondingly long relaxation time and, as a consequence, have a capillary number in the transitional regime. An alternative explanation is that the current model for bubble suspension rheology (equation (2.4)) is inadequate for non-dilute suspensions. That model can be derived from the theoretical treatment of Frankel & Acrivos [24] and Llewellin *et al.* [25], which is analytically exact in the limit of a dilute suspension (in which bubble–bubble interactions can be neglected) and in the limit of small bubble deformations. It is possible that bubble–bubble interactions in non-dilute samples act to introduce shear thinning—this would be consistent with our finding that shear thinning becomes more pronounced as bubble volume fraction increases. Further experimental work would be required to underpin a more detailed investigation of this phenomenon.

Superimposed on the decrease in flow index due to increasing bubble volume fraction is the effect of increasing particle volume fraction, shown most clearly in figure 10*b*. This relationship is nonlinear and appears to follow the empirical model proposed by Mueller *et al.* [19] (equation (2.14) with *r*_p_=1 for spherical particles). For bubble-free suspensions—comparable to those investigated by Mueller—there is excellent agreement between data and model for *ϕ*_p_/*ϕ*_m_<0.8, which is consistent with the limits of applicability given by Mueller *et al.* [19].

The combined effect of bubbles and particles on the flow index appears to be a simple superposition of these two effects: the flow index of a pure fluid is 1 and is reduced by some amount dependent on bubble volume fraction, and again by some amount dependent on particle volume fraction. Consequently, we propose the following purely empirical model for flow index for suspensions of spherical particles:

6.2 $$n=1-0.2{\left(\frac{\phi_{p}}{\phi_{m}}\right)}^{4}-0.334\phi_{b}.$$

**Figure 11.**
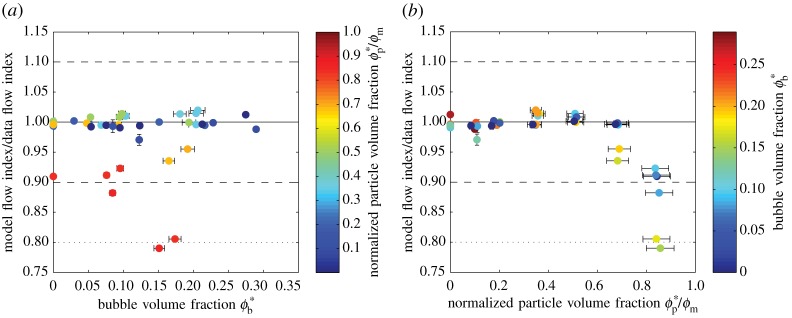
Comparison between *n* predicted by the model (equation (6.2)) and *n* calculated from experimental data, against (*a*) ϕ*_b_, shaded for ϕ*_p_/*ϕ*_m_ and (*b*) ϕ*_p_/*ϕ*_m_, shaded for ϕ*_b_. Error bars (1*σ*) are shown when larger than the data points. The solid line indicates a perfect match between model and data; the dashed and dotted lines, respectively, indicate ±10% and ±20% discrepancy. (Online version in colour.)

Curves of this model for various bubble volume fractions are shown in figure 10*b* and indicate that the model is valid for all samples with a total suspended fraction $\phi_{s}≲0.5$ (figure 11). The model agrees with the data to within ±5% for all samples below this cut-off. Note that this cut-off is drawn empirically from our data and has no theoretical basis.

For suspensions of non-spherical particles, the aspect ratio may be included in equation (6.2) in a manner analogous to equation (2.14). However, further experiments would be required to validate this extension.

## 7. Conclusion

Our results demonstrate that the proposed three-phase rheological model (equation (3.2)) based on an effective medium approach is in close agreement with experimental data over the full range $0\leq \phi_{b}^{*}≲0.3$ and $0\leq \phi_{p}^{*}/\phi_{m}≲0.85$ investigated. Our preliminary experiments involve spherical particles and steady flow in the low capillarity regime; hence, the model’s validity is only demonstrated subject to these restrictions. The model’s applicability is, however, potentially much broader (though extensions to the model require further experimental validation).

Mueller *et al.* [20] demonstrate that the Maron–Pierce relationship (equation (2.11)) is valid for suspensions of non-spherical particles when *ϕ*_m_ is calculated as a function of particle shape (equation (2.13)). Adopting this methodology for equation (3.2) broadens its applicability to natural systems, in which particles are rarely spherical.

The low-capillarity assumption can also be relaxed. Substituting equation (2.6) for equation (2.5) in the formulation of equation (3.2) yields a three-phase model suitable for high-capillarity flows. This version of the model predicts that the addition of bubbles will always reduce the reference viscosity of a three-phase suspension, even when particle concentration is low. At high particle concentrations, the reduction in viscosity is much more dramatic than in the low capillarity case.

Relaxing the assumption of steady flow is more challenging because, while the rheology of bubble suspensions in unsteady flow is well known [7,9], there is no adequate model for particle suspensions in unsteady flow.

The model developed in this study assumes that bubbles are small compared with particles, although the experimental data demonstrate that the model remains valid when bubbles and particles are comparable in size. Further experiments are required, however, to determine the rheology of suspensions in which bubbles are large compared with particles.

The proposed model for the flow index of three-phase suspensions (equation (6.2)) also provides good agreement with experimental data for which $\phi_{s}≲0.5$. Since this model is purely empirical, the relationship between the coefficients found here and the physical properties of the material is not clear: without a physical explanation for the occurrence of shear thinning in bubble and particle suspensions, more work is still needed.

## Supplementary Material

Supplementary Information

## Data accessibility

The full rheological data can be found online at the Earth Science Academic Archive, doi:10.5285/e729d509-a616-4787-bb41-44c1169685b6.
