# Supplementary material for: The rheology of three-phase suspensions at low bubble capillary number
Source: Proc Math Phys Eng Sci. 2015 Jan 8;471(2173):20140557. doi: 10.1098/rspa.2014.0557 (PMC4277193; doi:10.1098/rspa.2014.0557)
Supplement: Supplementary Information [file rspa20140557supp1.pdf]

Supplementary Information

1 Estimating the errors on fitted parameters

A number of important parameters for our study (η_*, n, ϕ_m, τ_0) are found by fitting equations to a set of experimental data. We can simply calculate a confidence limit on our fit to the data, but this does not account for errors on the experimental data. We use an adaptation of the bootstrapping technique for regression problems to estimate errors on the fit, which is described here in the context of fitting a value of ϕ_m to the Maron-Pierce relationship (equation 2.8), given a set of $\eta_{r,*}(\phi_p)$ data and errors:

ϕ_p	σ_p	$\eta_{r,*}$	σ_η
0.050	0.0015	1.2045	0.0096
0.100	0.003	1.4496	0.0103
0.200	0.006	2.2263	0.0122
0.300	0.009	3.9697	0.0226
0.400	0.012	9.6470	0.0685
0.500	0.015	40.3865	0.6462

Table 1: Experimental data and one standard deviation errors. These data appear in figure 4 of the main text.

If we assume that the errors on $\eta_{r,*}$ and ϕ_p are normally distributed, we can use each measured or calculated value as the mean, together with the known standard deviation error, to calculate a probability distribution for each value (e.g. figure 1). We can then resample the entire dataset, drawing values at random from each probability distribution, and use these values to calculate a best-fit ϕ_m. Repeating this process a very large number of times (10,000, in our case), results in a distribution of values of ϕ_m (figure 2).

Assuming that this distribution is normal, we can calculate a mean, $\phi_m = 0.5933$ (which is almost identical to the value calculated from a least squares fit to the original data, $\phi_m = 0.5934$), and a standard deviation, $\sigma_m = 0.0177$. Thus we have an estimate of the potential error in our value of ϕ_m.

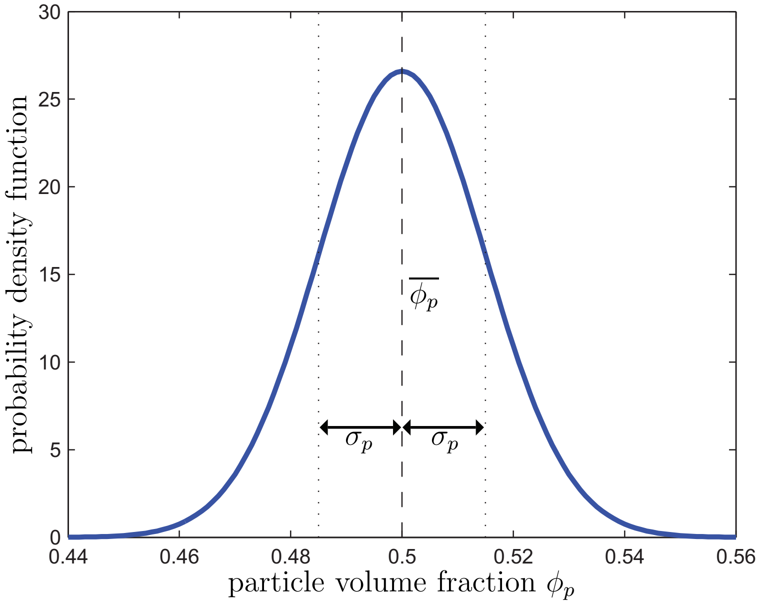

Figure 1: Probability density function for $\phi_p = 0.5$, given $\sigma_p = 0.015$.

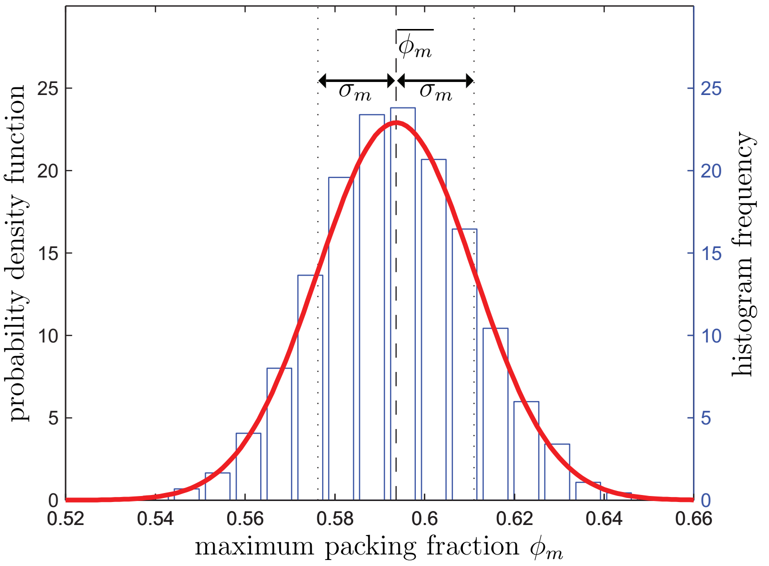

Figure 2: Histogram of fitted ϕ_m values. Red curve is the scaled probability density function, assuming that the ϕ_m values form a normal distribution.

2 Will increasing bubble volume fraction increase or decrease reference viscosity?

It is of interest to know the effect of adding an additional volume of bubbles to an existing suspension. Depending on the intial bubble and particle volume fractions, this may cause an increase in viscosity, a decrease in viscosity, or a decrease followed by an increase. At some point in between, the addition of an infinitesimally small volume of bubbles will cause no change in viscosity; this will be the points at which (from equation 3.2 in the main manuscript)

$$\frac{\partial \eta_{r,*}}{\partial \phi_b^*} = 0. \tag{1}$$

By finding the root of this equation, we can determine the location of the regime divide between bubbles causing an increase in viscosity, and bubbles causing a decrease in viscosity.

Expanded, the differential equation becomes

$$\frac{\partial \eta_{r,*}}{\partial \phi_b^*} = \frac{1-\phi_p^*}{\left(1-\frac{\phi_p^*(1-\phi_b^*)}{\phi_m}\right)^2\left(1-\phi_p^*(1-\phi_b^*)-\phi_b^*\right)^2} - \frac{2\phi_p^*}{\phi_m\left(1-\frac{\phi_b^*}{1-\phi_p^*(1-\phi_b^*)}\right)\left(1-\frac{\phi_p^*(1-\phi_b^*)}{\phi_m}\right)^3}, \tag{2}$$

and the root is

$$\phi_b^* = \frac{\sqrt{(9-8\phi_m)(\phi_p^*)^2} + 4(\phi_p^*)^2 - 3\phi_p^*}{4(\phi_p^*)^2}; \tag{3}$$

an explicit solution cannot be found for ϕ_p^*. Equation 3 is plotted in figure 3. For all suspensions with bubble and particle volume fractions to the left of the black curve, the addition of bubbles will lead to an increase in reference viscosity. An example is shown by the blue trajectory, for a fluid that begins with no suspended particles or bubbles. To the right of the black curve, addition of bubbles will initially lead to a decrease in reference viscosity. An example is shown by the red trajectory, for a fluid with an initial particle volume fraction $\phi_p^* = 0.5$. If enough bubbles are added that the suspension reaches the black curve, viscosity will begin to increase again. In order to determine the effect of adding some volume of bubbles to a suspension, both the initial bubble volume fraction and particle volume fraction must be known.

The effect of adding (or growing) bubbles, for an initially bubble-free suspension, is demonstrated clearly by our data (figure 4). For a particle-free suspension, addition of bubbles will always lead to a viscosity increase, compared to the bubble-free suspension (equivalent to the blue trajectory in figure 3); on the other hand, the addition of bubbles to a concentrated particle suspension leads to a viscosity decrease (equivalent to the red trajectory in figure 3).

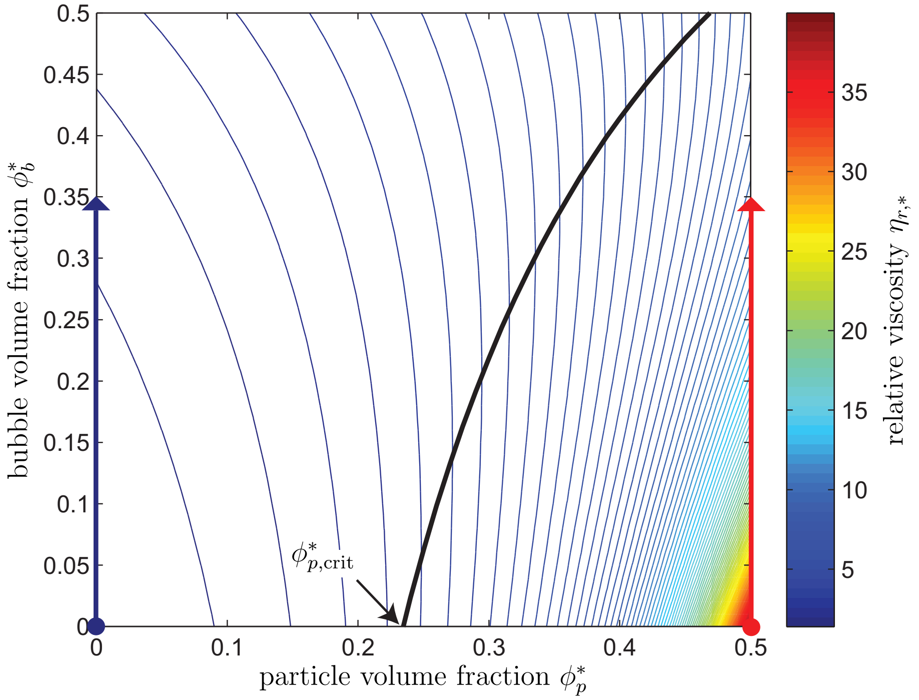

Figure 3: Contours of $\eta_{r,*}$ for ϕ_p^* and ϕ_b^*. The black curve is equation 3, and the point $\phi_{p,\mathrm{crit}}^*$ is the point at which addition of bubbles to an initially bubble-free suspension will switch from increasing to decreasing viscosity. The red and blue trajectories are described in the main text.

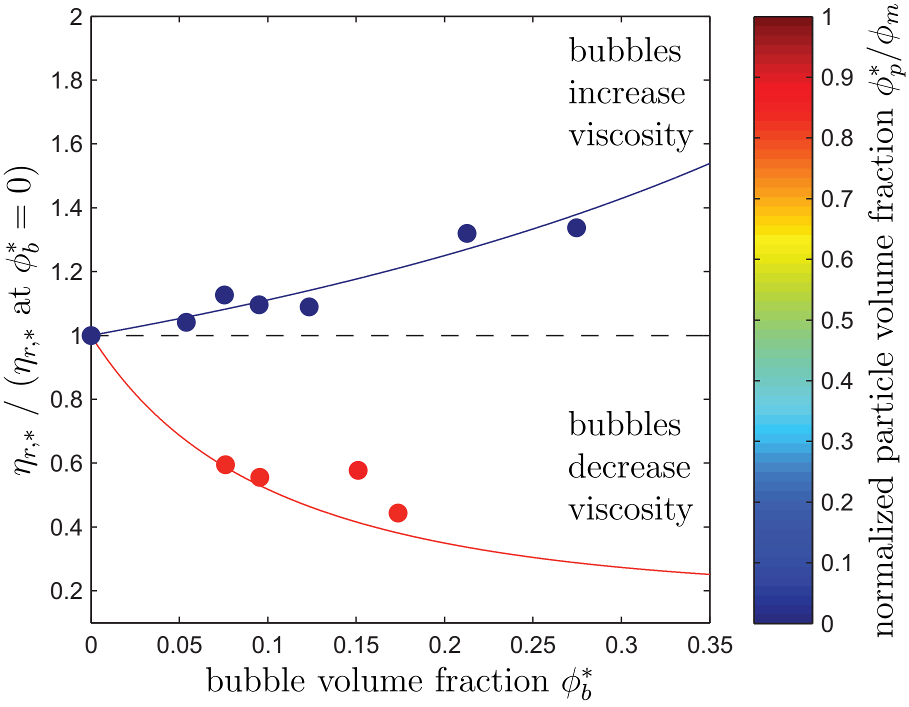

Figure 4: Relative viscosity $\eta_{r,*}$ normalised by the relative viscosity of the suspension with the same ϕ_p^* but no bubbles, i.e. $\eta_{r,*}$ / ($\eta_{r,*}$ at $\phi_b^* = 0$), for a particle-free suspension (blue datapoints and model curve) and a particle-rich suspension (red datapoints and model curve).
